# Does Mothers’ Self-Reported Mindful Parenting Relate to the Observed Quality of Parenting Behavior and Mother-Child Interaction?

**DOI:** 10.1007/s12671-020-01533-0

**Published:** 2020-11-10

**Authors:** Eva S. Potharst, Anna Leyland, Cristina Colonnesi, Irena K. Veringa, Eliala A. Salvadori, Marta Jakschik, Susan M. Bögels, Moniek A. J. Zeegers

**Affiliations:** 1 UvA minds, Academic Outpatient (Child and Adolescent) Treatment Center of the University of Amsterdan, Banstraat 29, 1071 JW Amsterdam, The Netherlands; 2 Research Institute of Child Development and Education, University of Amsterdam, Amsterdam, The Netherlands; 3 Department of Psychology, University of Sheffield, Sheffield, UK; 4 Developmental Psychology, University of Amsterdam, Amsterdam, The Netherlands

**Keywords:** Mindful parenting, Self-report measure, Observational measure, Maternal sensitivity, Mind-mindedness, Emotional communication, Mothers, Infants, Toddlers, Parent-child interaction, Parenting

## Abstract

**Objectives:**

Growing academic interest in mindful parenting (MP) requires a reliable and valid measure for use in research and clinical setting. Because MP concerns the way parents relate to, and nurture, their children, it is important to evaluate the associations between self-reported MP and observed parenting and parent-child interaction measures.

**Methods:**

Seventy-three mothers who experience difficulties with their young children aged 0–48 months admitted for a Mindful with your baby/toddler training (63% in a mental health care and 27% in a preventative context) were included. Mothers completed the Interpersonal Mindfulness in Parenting scale (IM-P) and video-observations of parent-child interactions were coded for maternal sensitivity, acceptance, mind-mindedness, and emotional communication (EC).

**Results:**

The IM-P total score was positively associated only with mothers’ gaze to the child (EC). IM-P subscale Listening with Full Attention negatively predicted non-attuned mind-mindedness, Compassion with the Child positively predicted maternal sensitivity and positive facial expression (EC), and Emotional Awareness of Self positively predicted mothers’ gaze to the child (EC) and dyadic synchrony of positive affect (EC).

**Conclusions:**

The current study provides support for the hypothesis that the IM-P total score is predictive of maternal actual attention for the child during a face-to-face interaction. When the IM-P is administered with the aim to gain understanding of different aspects of parenting behavior and the parent-child interaction, it is important not only to employ the IM-P total score but also to incorporate the individual IM-P subscales, as meaningful associations between IM-P subscales and observed parenting and parent-child interactions were found.

Since the mindfulness-based stress-reduction training (MBSR; Kabat-Zinn 1990) was developed for people experiencing high levels of stress, mindfulness-based interventions were adapted for a wide variety of application areas. For parents experiencing high levels of stress, mindful parenting (MP) programs were developed (e.g., Bögels et al. 2014; Singh et al. 2007) to reduce parents’ stress, increase child emotion regulation, and promote parenting and the quality of the parent-child relationship (Bögels et al. 2010; Duncan et al. 2009). MP is defined as the ongoing process of intentionally bringing moment-to-moment, non-judgmental awareness as best one can to the unfolding of one’s own lived experience, including parenting (Kabat-Zinn and Kabat-Zinn 1997). MP is shown to be associated with parenting stress, parenting style, parent-child relationship quality, and child well-being (Gouveia et al. 2016; Medeiros et al. 2016). A Portuguese study by Gouveia et al. (2016) showed that higher levels of MP were predictive of higher levels of authoritative parenting style, and lower levels of authoritarian, permissive parenting styles as well as reduced parenting stress. In an American study, it was found that MP mediated the negative relationship between parents’ general mindfulness and their children’s psychopathology (Parent et al. 2016). A Chinese study showed that parents’ general mindfulness was indirectly associated with child behavior problems through MP and through positive parenting practices (Han et al. 2019). Another Portuguese study showed a positive association between MP and security of the parent-child attachment relationship, which in turn was positively associated with child well-being (Medeiros et al. 2016). Hence, there is growing evidence showing the value of MP in developmental psychology and psychopathology (Moreira et al. 2019).

The interpersonal mindfulness in parenting (IM-P; Duncan 2007) has been developed in order to obtain a valid measure of MP. Duncan et al. (2009) introduced a model of MP, describing it as certain parenting skills and practices, namely the ability to maintain present-centered attention and awareness, to remain open, receptive, non-judgmental and compassionate towards both the self and the child, and to regulate emotions and impulses in parenting interactions. Based on this model, the interpersonal mindfulness in parenting (IM-P) scale was developed (Duncan 2007). In several countries, studies on psychometric properties of the IM-P were carried out (De Bruin et al. 2014; Kim et al. 2019; Lo et al. 2018, Moreira and Canavarro 2017; Pan et al. 2019). On the basis of a factor analysis performed in a Dutch study including mothers of adolescents from the general community, a 29-item and six-factor structure of the IM-P was proposed, with the following subscales: (1) Listening with Full Attention, (2) Compassion for the Child, (3) Non-judgmental Acceptance of Parental Functioning, (4) Emotional Non-reactivity in Parenting, (5) Emotional Awareness of the Child, and (6) Emotional Awareness of Self (De Bruin et al. 2014). Reliability of the total scale and four of the six subscales was good. Construct validity was also supported, as positive associations were found between the IM-P and aspects of general mindfulness, quality of life, and optimism, and negative associations with depression, parental overreactivity, verbosity, and laxness (De Bruin et al. 2014).

Subsequently, studies on the psychometric properties of the IM-P were carried out in general community samples of mothers and fathers in Portugal (Moreira and Canavarro 2017), Hong Kong (Lo et al. 2018), mainland China (Pan et al. 2019), and Korea (Kim et al. 2019). The age ranges of the children whose parents were included varied (one to 18-years old, two to 19 years old, four to 28 years old, and one to 18-years old, respectively), but no study examined parents of infants. Results showed inconsistencies in the factor structure of the IM-P across studies. Whereas internal consistency of the IM-P as a whole was good in all four studies, reliability of some of the subscales was not always satisfactory. The inconsistencies may either mean that the subscales are not reliable, or that the validity of the different subscales varies across different countries and cultures. The four studies showed positive correlations between the IM-P total scale and mindfulness and parental mental health measures. Negative correlations were found between the IM-P and dysfunctional parenting practices, parenting stress, and authoritarian and permissive parenting styles, and positive correlations between the IM-P and parental warmth and an authoritative parenting style (Kim et al. 2019; Moreira and Canavarro 2017; Pan et al. 2019). However, all these parenting-related outcomes were measured using questionnaires, and measuring aspects of parenting and parent-child interaction using self-report measures has limitations (Miron et al. 2009).

There are two main challenges in capturing the complexity of the relational dynamics of parenting and parent-child interaction with self-report questionnaires (Morsbach and Prinz 2006). First, bias may be caused by misinterpretations and misunderstanding of certain questions (Morsbach and Prinz 2006). Second, especially when questionnaires are directed at sensitive issues, which is also the case for the parenting behavior and the parent-child relationship, participants tend to answer questions in a socially desirable way (Morsbach and Prinz 2006). In fact, Herbers et al. (2017) showed that agreement between self-report and observational measures (especially those focused on negative parenting behaviors) was lower for parents with higher levels of distress and lower levels of socioeconomic status (SES).

Due to the importance of validating IM-P with more objective parenting and parent-child interactions, some studies have employed observational measures (Duncan et al. 2015; Turpyn and Chaplin 2016). In a study by Turpyn and Chaplin (2016) in a community sample of 157 primary caregivers and their 12- to 14-year-old adolescents, the 10-item version of the IM-P was completed by the parents, and the Parent-Adolescent Interaction Task was used to measure observed parent’s positive and negative emotions, and the parent-child dyad’s shared positive emotion during a conflict situation between the parent and the adolescent. The IM-P was negatively associated with parents’ observed negative emotion and positively associated with shared positive emotion. In a study by Duncan et al. (2015) in a community sample of 375 mothers and their 12- to 13-year-old adolescents, also the 10-item version of the IM-P was completed by parents, and the Iowa Family Interaction Rating Scales were used to measure observed maternal parenting, and dyadic interactions. The IM-P was associated with less harsh and more positive parenting, consistent discipline, communication skills, maternal warmth, and positive interaction.

As the only studies on the relation between the IM-P and observed parenting measures focused on parents with adolescents, it remains unclear whether the IM-P is also associated with observed quality of parenting and parent-child interaction in samples with younger children, such as infants and toddlers. This age group is especially relevant because parenting behavior and parent-child relationship, especially in the first years of life, influences the child’s emotional, social, and cognitive development (e.g., Bernier et al. 2010). Because understanding the role of MP offers possibilities for intervention (Bögels et al. 2010; Duncan et al. 2009), it is especially important that MP can be measured in a valid way in parents of young children who experience problems in parenting and in the parent-child interaction. In order to assess the external validity of the IM-P for this specific group, we need to test associations between the IM-P and well-established observational measures that are thought to be crucial to developing parent-child relationships.

The most observed parenting dimension in early childhood is sensitivity (Ainsworth 1969), which refers to the parent’s ability to understand the child’s signals, appropriately and promptly responding to them. The construct of parental sensitivity was advanced to explain the development of secure attachment behavior in young children and has been repeatedly established as a predictor of infant-parent attachment security (e.g., De Wolff and Van IJzendoorn 1997), and many other positive outcomes in young children, such as affect regulation (e.g., Braungart-Rieker et al. 2001), social functioning (e.g., Kochanska 2002), and better performance in cognitive or language-related tasks (e.g., Tamis-LeMonda et al. 2001). Because MP involves being attentive to the child, and open to what the child is showing and saying, we may expect parents scoring high on MP to show greater sensitivity to their child’s cues during interactions. Another parenting concept that may be related to MP is parental acceptance of the child, which means that the parent respects the child’s autonomy and is able to accept expressions of initiative and emotion, maintaining sufficient balance between positive and negative feelings towards the child (Ainsworth 1969). Parental acceptance of the child’s emotions has a predictive value of later child emotion regulation and coping (Kliewer et al. 1996). As respect of autonomy, acceptance of (the difficult feelings of) the child, compassion for the child, and good self-regulation of emotions that arise in the parent-child relationship are important elements of MP, it is reasonable to expect that high IM-P scores predict parental acceptance of the child.

An additional important established parental predictor of positive development in early childhood is mind-mindedness, which can be described as parents’ tendency to treat their child as a mental agent, and thus as an individual with autonomous thoughts, feelings, and desires (Meins 2013). Mind-mindedness may also be viewed as a measure of parents’ mentalizing tendency (Sharp and Fonagy 2008), and it is assessed by considering parents’ (appropriate or non-attuned) comments on their child’s presumed internal states during interactions (Meins and Fernyhough 2015). Mind-related comments are appropriate when they actually reflect the child’s mental state, while with non-attuned mind-related comments the parent misreads the internal state of the child. Parental mind-mindedness has shown to be an independent predictor of secure parent-infant attachment, over and above parental sensitivity (Zeegers et al. 2017). Particularly, parents’ frequent misreading of their child’s behavior and mind (i.e., non-attuned mind-mindedness) has shown to predict insecure attachment and later externalizing problems and low social competence in children (Colonnesi et al., 2019; Zeegers et al., 2017). Since MP reflects parental awareness of the child’s own emotions, whether they are similar or different from their own, higher IM-P scores may be associated with mothers’ mind-mindedness.

A different way of assessing the quality of the parent-child interaction is by observing the moment-to-moment dynamics of face-to-face interactions between infants and their mothers (Yale et al. 2003). Attention to (gaze at) the social partner, positive and negative facial expressions, and verbalizations (*n* of seconds) can be coded as distinct behaviors occurring during the emotional communication. In addition, dyadic synchrony is characterized by the temporal co-occurrences of the same behavior displayed by both interaction partners (e.g., the mother smiles to the child while the child smiles to the mother; Colonnesi et al. 2012; Yale et al. 2003). A high level of dyadic synchrony implies that the parent is responsive to the child and that there is reciprocity between mother and child. A low level of dyadic synchrony between a mother and infant could represent a risk factor for the child’s later social-emotional problems (Leclère et al. 2014). Parents scoring high on the IM-P are expected to be more attentive to their child (being able to keep their eyes focused on the child), to display more positive facial expressions, avoid negative reactions, and to verbalize more often to the child. Also, mindful parents are expected to be more synchronized with the positive facial expressions of the child, reproducing the same expressions, thus engaging in longer episodes of dyadic positive affects with their child.

The current study examines the associations between mothers’ self-reported MP and observed parenting behavior in mother-child interactions with children aged 0 to 4 years old. We hypothesized that the IM-P total score would relate to different aspects of parenting and mother-child interaction. More specifically, we assessed the relations between the IM-P total scores and (a) parental sensitivity and acceptance of the child, (b) parental appropriate and non-attuned mind-mindedness, and (c) maternal emotional communication (i.e., gaze, positive facial expression, verbalization) and dyadic synchrony (i.e., temporal co-occurrence of the parent and the child positive facial expression). Lastly, to get a better idea of the external validity of the individual subscales of the IM-P, we explored whether there were any specific relations between the 6 subscales (i.e., (1) Listening with Full Attention, (2) Compassion for the Child, (3) Non-judgmental Acceptance of Parental Functioning, (4) Emotional Non-reactivity in Parenting, (5) Emotional Awareness of the Child, and (6) Emotional Awareness of Self (De Bruin et al. 2014) and the observed parenting and parent-child interaction constructs.

## Method

### Participants

The mothers in the current study (*N* = 73) also participated in (published and ongoing) studies examining the effectiveness of the “Mindful with your baby/toddler” interventions (Potharst et al. 2017; Potharst et al. 2018; Zeegers et al. 2019). Forty-six mothers and their children (63%) participated in the training in a mental health context. These mothers had been referred to the training for reasons like maternal mental health problems, child regulation problems, or parent-child interaction problems. Twenty-seven (37%) of the mothers and their children participated on their own initiative, in a preventative context. These mothers wished to take part because they wanted, for example, to learn to better deal with parenting stress, take better care of themselves as parents or improve the contact with their children. For one mother, socio-demographic information was unavailable. Percentages were calculated on the basis of the remaining 72 participants. Thirty-eight mothers (52%) received psychological or pedagogical treatment prior to the training. During the intake that took place before the training, mothers reported to the trainer symptoms of depression (*N =* 26; 36%), anxiety disorders (*N =* 19; 26%), and post-traumatic stress disorder (*N =* 8; 11%). The majority of the participants had at least one of these mental health problems (41; 57%).

Fifty-two mothers (71%) were admitted to the Mindful with your baby training and had infants aged 0 to 18 months, and 21 mothers (29%) were admitted to the Mindful with your toddler training and had toddlers between the age of 18 and 48 months. The mean age of the mothers was 35.2 years (*SD* = 4.2), and the mean age of the children 1.3 years (*SD* = 1.00; range .12–3.86 years). Forty-two of the children were boys (57.5%), and 54 were firstborns (74%). Thirty-eight (52%) mothers had a job [5 (7%) full-time, and 33 (45%) part-time], seventeen (23%) mothers were on sick leave, 4 (6%) mothers were on parental leave, and thirteen (18%) mothers chose to stay at home with their child and not to work. Employment status of one mother (1%) was unknown. Level of education was high in 61 (84%) mothers, low to moderate in 11 (15%) mothers, and unknown in one mother (1%). The ethnical background of was Dutch in 56 (78%) of the mothers, and the remaining as follows: Surinam (3, 4%), Turkish (2; 3%), Moroccan (2; 3%), Polish (2; 3%), German (2; 3%), Indian (1; 1%), Spanish (1; 1%), Eritrean (1; 1%), Italian (1; 1%), American (1; 1%), Romanian (1; 1%).

### Procedure

The data for the current study were retrieved from datasets of studies examining the effectiveness of the Mindful with your baby and Mindful with your toddler training. The starting dates of the trainings were between October 2015 and May 2020 and took place in Amsterdam and in The Hague. Only data that was gathered before participation in the training was used in the current study, namely in either the waitlist assessment or the pretest assessment. Whereas the pretest assessment was administered to all participants in the week prior to the start of the training, the waitlist assessment was administered only in case participants were admitted more than 5 weeks prior to the training. Data from the first available measurement occasion was used, unless the pretest assessment was more complete than the waitlist assessment. The design of the study consisted of a home-visit to video record a 10-min free-play session and a 4-min face-to-face parent-child interactions, as well as several online questionnaires. For the current study, only the IM-P questionnaire was used. For the face-to-face interaction observation, a two-sided camera was employed to capture both the mother’s and the child’s face and upper body. In the period between March and May 2020, home visits were not possible because of the measures that were taken in prevention of the COVID-19, and thus, video-observations were collected online (*N* = 8; 11%). Participants were only included in the current study if they had completed the IM-P, participated in the video-observations, and spoke Dutch with their child in the video-observation.

### Measures

#### Mindful Parenting

To measure MP, the Dutch version (De Bruin et al. 2014) of the Interpersonal Mindfulness in Parenting scale (IM-P; Duncan 2007) was used. The 29 items were scored on a 5-point Likert scale, ranging from 1 (never true) to 5 (always true), where higher scores indicate that mothers are more mindful in their parenting. In a Dutch validation study, a factor analysis revealed a structure of six dimensions: Listening with Full Attention, Compassion for the Child, Non-judgmental Acceptance of Parental Functioning, Emotional Non-reactivity in Parenting, Emotional Awareness of the Child, and Emotional Awareness of Self, the first five of which showed satisfactory reliability (De Bruin et al. 2014).

For mothers with infants between 0 and 18 months, small adaptations were made in the formulation of the items, for example, “child” was changed into “baby” and “parenting” or “raising” to “nurturing.” Furthermore, of the 29-item questionnaire, four items (item 4, 7, 8, and 28) were eliminated as they did not seem applicable for mothers of an infant, resulting in differently calculated scores between mothers of toddler and mothers of babies for the total score, Compassion for the Child, and Emotional Awareness of Self subscales. To make baby and toddler scores comparable, mean scores were used instead of sum-scores. Internal consistency was also calculated separately for these (sub)scales. Cronbach’s alpha of the total score was .87 for mothers with a baby, and .91 for mothers with a toddler, of the Compassion for the Child subscale .62 and .75, and for the Emotional Awareness of Self subscale .65 and .68, respectively. The other subscales, Listening with Full Attention, Non-judgmental Acceptance of Parental Functioning, Emotional Non-reactivity in Parenting, and Emotional Awareness of the Child had Crohnbach’s alpha’s of .86, .78, .65, and .80. For subscales with relatively weak internal consistency (α < .70), it was checked whether reliability could be improved by deleting items, while maintaining a minimum of three items. This was only possible for subscale Emotional Non-reactivity in Parenting, but not for the Compassion for the Child for mothers with a baby or the Emotional Awareness of Self subscale. Item 10 and 11 were deleted from subscale Emotional Non-reactivity in Parenting, which resulted in an alpha of .71. The deleted items were also removed from the total score.

#### Sensitivity and Acceptance

Maternal sensitivity and acceptance were observed during the 10-min free-play sessions. Mothers were instructed to play with their child with (5 min) and without (5 min) age-appropriate toys. Sensitivity and acceptance were assessed using the scale descriptions of Ainsworth (1969). The scale sensitivity captured whether a mother was sensitive or insensitive to the signals of her child. Sensitive mothers made themselves available to perceive child signals, attributed meaning to these signals by acting promptly and appropriately upon them. The acceptance scale captured whether a mother showed acceptance of the child’s initiatives and positive and negative feelings, showing patience, positive affectivity, and warmth towards the child. Video-observations were coded by five trained coders who were blind to the measurement occasion. Eighteen percent of the observations were coded to assess inter-rater agreement. The intra-class correlation among the independent coders was good (ICC = .83) for the sensitivity versus insensitivity scale and good (ICC = .76) for the acceptance versus rejection scale (Cicchetti 1994). After satisfactory ICC between the coders had been established, every video was coded twice, by two different observers. Differences in scores were resolved by discussion.

#### Mind-Mindedness

Mothers’ mind-mindedness was observed on the basis of the same 10-min free-play session that was used to assess maternal sensitivity and acceptance. Each spoken word or sentence of the mother was transcribed and coded by independent observers using a Dutch version of the mind-mindedness coding manual (Meins and Fernyhough 2015). The mind-related comments were categorized according to the specific state the parent referred to. Categories were *cognitions* (e.g., “you recognize this toy from the daycare center”), *likes and dislikes* (e.g., “you don’t like this book”), *emotions* (e.g., “you’re excited to play with these toys”), and *epistemic states* (i.e., “are you teasing me?”). Comments that were obviously meant to be dialog said/thought by the infant (e.g., “Mommy, pick me up please”) were also classified as mind-related.

Second, mind-related comments were classified as being appropriate or non-attuned. Appropriate comments are those for which: (a) the trained coder agreed with the parent’s reading of the child’s internal state, (b) the internal state comment linked the child’s current activity with similar events in the past or future, or (c) the parent voiced (using the first person) what the child might say if he or she could speak. Comments were classified as non-attuned when the coder believed (a) the parent misread the internal state of the child, or (b) the comment referred to a past or future event that had no obvious relation to the infant’s current activity (e.g., “I’m sure you would like to see grandma tomorrow”). In order to control for maternal verbosity, we calculated proportions of mind-related comments by dividing the total amount of appropriate or non-attuned comments by the total amount of comments a mother made during the free-play session (Meins and Fernyhough 2015). Twenty percent of the observations were randomly selected to calculate the inter-rater agreement. The inter-rater agreement was *α* = .96 for mind-related comments and *α* = .81 for appropriateness of mind-related comments, which can be classified as “almost perfect agreement” and “good agreement” (Landis and Koch 1977). Disagreements were resolved by discussion.

#### Emotional Communication and Dyadic Synchrony

In order to capture mothers’ emotional communication and parent-child dyadic synchrony, a four-minute face-to-face interaction was observed (Colonnesi et al. 2012). The child was placed in a seat in front of the mother (keeping a 30 to 50-cm distance), and the mother was instructed to talk to and play with her child, as she would normally do. A dual-lens camera recorded both the mother’s and the child’s face. Four trained observers coded second-by-second the videos with respect to mothers’ singular behaviors (gaze direction, facial expressions, and verbalizations) and children’s facial expressions. Behaviors were coded as state events (event with a start time and an end time), and the time-based co-occurrences of dyadic synchrony were calculated, using the software *The Observer XT 13.0* (Noldus et al. 2000; Zimmerman et al. 2009).

The coding for mother’s gaze included (a) gaze at the child when mothers were looking at their children’s face or hands, and (b) gaze otherwise referred to mothers looking away, and c) non-observable looking when the mothers’ face was not in the frame. Gaze otherwise was not included in the further analysis, but it represents the remaining time of the observation (240 s). The emotional valence of mothers’ and children’s facial expressions was coded as (a) positive, or (b) neutral or (c) negative. Positive facial expressions were identified with closed and open smiles, defined by raising corners of the lips (Colonnesi et al., 2012; Ekman and Friesen 1978). Verbalizations included talking (words or sentences) and vocalizations: positive vocalizations such as chuckling, giggling, or laughing, neutral vocalizations such as babble, and negative vocalizations such as crying or fussing. For the analyses, positive and negative vocalizations were added up to a total vocalization score. Vegetative and reflexive vocalizations (hiccups, coughs, burps, etc.) were not coded. The inter-rater agreement of mother’s gaze, facial expressions, and vocalizations could be classified as ‘almost perfect’ (*κ =* .88, *κ =* .89, and *κ =* .87, respectively). Proportions of mother’s gaze at the child, positive facial expressions, and verbalizations were based on the duration of the codable observation (i.e., frames during which the mother’s or child’s face was not visible were subtracted from the total duration of the observation). For the present study, only the proportion of dyadic synchrony (i.e., temporal co-occurrence in seconds) of mother’s and child’s positive facial expressions was used (Riehle et al. 2017).

### Data Analyses

Missing inspection revealed that 1.5% of the IM-P items was missing. It was therefore chosen to use mean subscale scores instead of sum subscale scores, so that it was still possible to calculate all subscale scores for all participants. Inspection of variable distributions indicated sufficient normality; skewness and kurtosis of all variables were < |2|, except for appropriate mind-related comments, and mother’s gaze. Two, and two outliers (*Z* > |2.58|) respectively were replaced by the next most extreme value at the end of the distribution of this variable (Field 2009). Correlation analyses were performed to assess the associations between the IM-P and its subscales and parental behavior and mother-child interaction outcomes. To test whether dimensions of MP (IM-P subscales) predicted observational measures of mother-child interactions (sensitivity/acceptance, mind-mindedness, emotional communication/dyadic synchrony) linear multiple regression analyses were conducted using a deletion approach, which consists in initially testing a full model containing all predictors, and then sequentially deleting in subsequent models those predictors that make the least contribution to the model (Tabachnick et al. 2019). In the interest of parsimony, for each outcome, the model that contained the fewest predictors was selected [non-significant predictors with *p >* .1 were removed] but only where there was no significant change in the predictive power (*p* F Δ R^2^ < .05) of the model. To ensure comprehension, data from the full model and the selected model were reported. Only significant models or borderline significant models containing at least one significant predictor were fully reported in the Results section. Prior to running the main analyses, the degree to which residuals met the assumptions of multivariate analysis were assessed through statistical tests and graphical representations. Mahalanobis distance scores indicated no multivariate outliers. Preliminary analyses revealed violation of the assumptions of homoscedasticity for one outcome: mother’s gaze. Therefore, for this outcome variable weighted least squares regression analysis was used instead of linear regression analysis (Field 2009). No multicollinearity was found. For the full models, the highest VIF was 1.76 (average 1.51), 1.60 (average 1.47), and 2.48 (average 1.78) for the Sensitivity/Acceptation, mind-mindedness, and the emotional communication models, respectively. For the only final model containing > 1 predictor (Sensitivity), the average VIF was 1.1.

## Results

Numbers on the available data (*n*) and descriptive statistics (*M*, *SD*) of independent (IM-P total score and subscale scores) and dependent variables (maternal parental behavior and mother-child interaction scores) are shown in Table 1. IM-P scores and mind-mindedness scores were available for every participant, and sensitivity/acceptance scores for almost 90% of the participants. The emotional communication/dyadic synchrony outcomes were only available for half of the participants, due to technical difficulties (such as unclear recordings or the unavailability of a two-sided video-camera) or due to observations that were done online because of COVID-19. Correlations among experimental variables are shown in Table 2. The total IM-P score showed a positive and significant association with one of the outcome measures: mother’s gaze to the child.

**Table 1:** Descriptive statistics of the independent variables (IM-P total score and subscale scores) and dependent variables (maternal parental behavior and mother-child interaction scores)

Variable	*n* (% of *n* = 73)	*M* (*SD*)
Mindful parenting (IM-P total score)	73 (100%)	3.3 (.5)
Listening with full attention		3.1 (.7)
Compassion with the child		4.4 (.5)
Non-judgmental acceptance of parental functioning		2.7 (.7)
Emotional non-reactivity in parenting		3.7 (.8)
Emotional awareness of the child		3.4 (.7)
Emotional awareness of the self		3.5 (.8)
Ainsworth Sensitivity Scales	64 (88%)	
Sensitivity		5.5 (1.7)
Acceptance of the child		6.0 (1.9)
Mind-mindedness	72 (99%)	
Appropriate mind-related comments		.06 (.03)
Non-attuned mind-related comments		.02 (.02)
Emotional communication	36 (49%)	
Mother’s gaze		96.0 (4.0)
Mother’s positive facial expression		47.2 (26.3)
Mother’s verbalization		58.4 (17.6)
Mother-child dyadic synchrony of positive facial expression		15.6 (12.8)

Data are presented as mean (standard deviation)

**Table 2:** Pearson’s correlations of the independent variables (IM-P total score and subscale scores) and dependent variables (maternal parental behavior and mother-child interaction scores)

	1	2	3	4	5	6	7	8	9	10	11	12	13	14
1. IM-P total														
2. LFA														
3. CC		.34**												
4. NJAPF		.42***	.17											
5. ENRP		.29*	.38**	.32**										
6. EAC		.42***	.28*	.52**	.17									
7.EAS		.28*	.47***	.32**	.37**	.33**								
8. Sensitivity	− .01	− .15	.20	− .06	− .01	.03	.15							
9. Acceptance	.08	.02	.24^†^	− .04	.11	− .01	.14	.80***						
10. Appropriate MRC	− .10	− .09	− .13	.03	− .06	− .19	− .13	.14	.09					
11. Non-attuned MRC	− .10	− .26*	− .03	− .06	− .07	− .07	.08	.03	− .11	.31**				
12. Mother’s gaze	.33*	.27	.35*	.04	.13	.39**	.35*	.30^†^	.31^†^	− .05	− .06			
13. Mother’s positive FE	.03	− .14	.33^†^	− .07	.26	.01	.08	.54**	.47**	.03	.03	.42*		
14. Mother’s verbalization	.16	.01	.19	.17	.00	.07	.30^†^	.31^†^	.29^†^	.12	.23	.30^†^	.18	
15. Dyadic synchrony of positive FE	.30^†^	.06	.26	.12	.12	.21	.41*	.57**	.53**	.08	− .02	.35*	.71***	.34*

*IM-P*, Interpersonal Mindfulness in Parenting Scale; *LFA*, Listening with full attention; *CC*, Compassion for the child; *NJAPF*, Non-judgmental acceptance of parental functioning; *ENRP*, Emotional non-reactivity in parenting, *EAC*, Emotional awareness in the child; *EAS*, emotional awareness in the self; *MRC*, mind-related comments; *FE,* facial expression

^†^*p* < .10, *p < .05, **p < .01, ***p < .001

### Mindful Parenting as a Predictor of Observed Parent-Child Interaction

The findings from the main regression analyses testing whether IM-P subscales predicted parental behavior and mother-child interaction are reported in Table 3.

**Table 3:** Backward entry linear regression models for outcomes of parental behavior and mother-child interaction outcomes by dimensions of mindful parenting (IM-P subscales)

	Sensitivity		Acceptance		Appropriate MRC		Non-attuned MRC		Mother’s gaze ^a^		Mother’s positive FE		Mother verbalizations		Dyadic synchrony of positive FE	
Model^b^	1	5	1	6	1	6	1	6	1	6	1	6	1	6	1	6
*R*^2^	.11	.09	.08	.06	.07	.04	.10	.07	.35	.26	.23	.11	.17	.09	.24	.17
*F*	1.17	2.93	0.77	3.75	.85	2.78	1.18	5.12	2.63	11.72	1.47	4.12	.95	3.45	1.51	6.79
*p*	.337	.061^†^	.598	.057^†^	.534	.100^†^	.327	.027*	.037*	.002**	.224	.050*	.474	.072^†^	.211	.014*
*f*^2^	.12	.10	.09	.06	.08	.04	.11	.08	.54	.35	.30	.12	.20	.10	.32	.20
*F* Δ *R*^2^		.91		.63		1.60		2.01		.864		2.60		.86		.48
*p F* Δ *R*^2^		.345		.430		.210		.161		.359		.116		.361		.494
Standardized *β* IM-P-subscales																
LFA	− .25	− .23^†^	− .03		− .02		− .30*	− .26*	− .16		− .32		− .12		− .25	
CC	.22	.27*	.23	0.24^†^	− .05		− .01		.10		.31	.33*	.20		.08	
NJAPF	− .06		− .06		.21		.04		− .23		− .25		.18		− .24	
ENRP	− .07		.04		− .04		− .06		− .20		.25		− .24		.11	
EAC	.07		− .08		− .24	− .19^†^	− .01		.29		.17		− .12		.27	
EAS	.15		.08		− .08		.19		.58*	.51**	.01		.35	.30^†^	.42*	.41*

*IM-P*, Interpersonal Mindfulness in Parenting Scale; *LFA*, listening with full attention; *CC*, compassion for the child; *NJAPF*, non-judgmental acceptance of parental functioning; *ENRP*, emotional non-reactivity in parenting, *EAC*, emotional awareness in the child; *EAS*, emotional awareness in the self; *MRC*, mind-related comments; *FE*, facial expression; *NA*, negative affect

^a^Output from weighted least squares regression

^b^Six regression models were run for each outcome using a backwards deletion process (criteria out *p* < .10), the model numbers in this row refer to 1 the full model (all six predictors) and then the selected model number (retaining 1–5 predictors)

^†^*p* < .10, *p < .05, **p < .01

#### Sensitivity and Acceptance

IM-P predicted 9–11% of variance of maternal sensitivity and 6–8% of variance of maternal acceptance. The final model for maternal sensitivity was non-significant, but did contain a significant predictor, namely IM-P subscale Compassion for the Child (positive relation), and a non-significant predictor, namely subscale Listening with Full Attention (negative relation). This model explained 9% of the variance of sensitivity (small effect size). The final model for maternal acceptance of the child was non-significant and did not contain significant predictor variables.

#### Mind-Mindedness

MP predicted only a small amount of variance in mind-mindedness; 0–7% of Appropriate mind-related comments and 7–10% of Non-attuned mind-related comments. The final model for appropriate mind-related comments was non-significant and did not contain significant predictor variables. The final model for non-attuned mind-related comments was significant, predicted 7% of variance of the outcome (small effect size), and contained one IM-P subscale: Listening with Full Attention (significant negative relation).

#### Emotional Communication and Dyadic Synchrony

MP predicted 26–35% of variance in mother’s gaze, 11–23% of variance in mother’s positive facial expression, 9–17% of variance in mother’s verbalization, 17–24% of variance in dyadic synchrony of positive facial expression, and 8–12% of variance in outcomes of maternal responsiveness to child’s negative affect. The final model for mother’s gaze was significant, predicted 26% of variance of the outcome (large effect size), and contained one IM-P subscale: Emotional Awareness of Self (significant positive relation). The final model for mother’s positive facial expression was significant, predicted 11% variance of the outcome (small effect size) and contained one IM-P subscale: Compassion for the Child (significant positive relation). The final model for mother’s verbalization was non-significant and did not contain significant predictor variables. The final model for dyadic synchrony of positive facial expression was significant, predicted 17% of variance (medium effect size) and contained one IM-P subscale: Emotional Awareness of Self (significant positive relation).

### Post hoc Power Calculations

A post hoc power analysis was conducted using the software G*Power (Faul et al. 2007). For the calculation, the 6-variables models were used and an alpha error probability of .05 was selected. Furthermore, we used the mean effect size that we have found in the current study (*f*^2^ = .22). With a sample size of 73, a power of .83 was revealed. This means that for analyses in which an effect size of *f*^2^ = .22 was shown, and 73 participants were included, power was acceptable. It should be noted, however, that in the analyses showing higher effect sizes (emotional communication/dyadic synchrony) sample size was smaller, and in the other analyses, effect sizes were smaller.

## Discussion

The present study examined the association between self-reported mindful parenting (IM-P total score and subscales) and observed parenting behavior and mother-child interactions in dyads with children 0 to 4 years old. The only outcome measure that the IM-P total score was significantly and positively associated with was mothers’ gaze to the child, indicating attention to the child’s face. This means that in the second-by-second coded face-to-face interaction between mothers and their children, mothers scoring high on the IM-P were those spending more time paying attention to their child. Linear multiple regression models were used to test which IM-P subscales predicted the observational outcomes. In the model predicting maternal sensitivity, subscale Compassion for the Child was the only significant predictor, while no significant predictors were found for outcome maternal acceptance of the child. This was also the case for outcome appropriate mind-related comments, and for non-attuned mind-related comments, subscale Listening with Full Attention was shown to be a significant predictor. With regard to other’s gaze and dyadic synchrony of positive facial expressions, subscale Emotional Awareness of Self was shown to be a significant predictor, and Compassion for the Child significantly predicted mothers’ positive facial expressions. No significant predictor was found for mother’s verbalizations.

Overall, the results of this study provide partial evidence to the hypothesis that the IM-P total score predicts different aspects of observed parenting behavior or mother-child interaction. This result finds support in prior studies reporting low association between self-reported parenting behavior and observed parenting behavior (Arney 2004; Bennetts et al. 2016). Reasons for this low concordance between self-reported and observed parenting may be related to low reliability of (one of) the measuring methods. Specific problems that may play a role in self-report are social desirability bias, and difficulties with recognizing and recalling own parenting behavior (Arney 2004). Specifically with regard to mindfulness in parenting, it could be that parents who are more mindful may sometimes score lower because they have more awareness of the difficult parenting moments and the moments they are not mindful with their children. Other problems arise in observational measures, namely that these are, in comparison with self-report that covers many behaviors in many situations, context-specific, and may be influenced by the presence of the observer (Gardner 2000). Notably, also, the observed measures that were used in this study did not have the aim to measure mindful parenting. Moreover, the clinical character of the sample, that is, mothers who are referred or self-refer to a mindful parenting training because they experience difficulties in parenting their child, may have lowered the associations between self-reported and observed parenting. Herbers et al. 2017 showed other characteristics that may moderate the relation between self-reported and observed parenting: concordance between the measurement methods was lower for parents with higher levels of parent distress (symptoms of depression) and a lower level of SES. Parents who have more stress and/or mental health problems also have more difficulties mentalizing about themselves and their relationships (Fearon et al. 2006). In the current group, more than half of the mothers had mental health problems, which may have played a role in the outcome that the IM-P total score did not correlate significantly with most of the outcomes. The fact that our sample concerned only mothers of infants and toddlers does not appear an explanation for the limited associations we found between self-reported mindful parenting and observed parenting, as associations between self-reported and observed parenting behavior seems to be higher for parents with preschool-aged children than for parents with older children (Arney 2004).

The only outcome that the IM-P total score was significantly associated with was the duration of mother’s gaze to their child during the face-to-face interaction. Mother’s gaze is a measure of (visual) attention, and attention for the child is a prerequisite for responsiveness (Kim et al. 2014). By directing her gaze at the child, the mother shows her attention, interest, and availability to the child, supporting the child in communicating with her. This finding was expected, as a core feature of MP is in fact being attentive (Duncan et al. 2009). The objective measure of mother’s gaze confirms that mothers who perceive themselves as attentive parents are indeed more attentive when interacting with their children.

When exploring which of the specific dimensions of MP accounted for the association between the IM-P total score and mother’s gaze, it was shown that Emotional Awareness of Self was the only significant predictor. Emotional Awareness of Self was also a significant predictor of parent-child dyadic synchrony of positive facial expression (medium effect size), which means that mothers scoring high on Emotional Awareness of Self had longer moments of simultaneous positive facial expressions with their child. This result may at first seem surprising, as both in mother’s gaze and dyadic synchrony of positive facial expression an essential ability is to be present with and focus on the child instead of on the self. However, maternal emotional awareness of self may be at the basis of the capacity to also be emotionally aware of the other, and in this case the child. Luyten et al. (2017) wrote that “parental reflective functioning (the capacity to treat the child as a mental agent) is thought of as a relationship-specific manifestation of the more general capacity for reflective functioning” (p. 2). The awareness of emotions leads to decentering (being able to observe a feeling with some distance without judging it, and knowing it will pass), which prevents a parent from giving emotionally driven automatic reactions, and supports the parent in staying present with their child and making conscious decisions in parenting (Duncan et al. 2009; Sauer and Baer 2010). This means that the awareness of the inner experience influences the behavior of the parent towards the child. This is also apparent in the formulation of the items of the subscale Emotional Awareness of Self: all items include both awareness of the difficulty or the emotional reaction of the parent and the reaction of the parent to the child (e.g., item 21: “In difficult situations with my child, I pause without immediately reacting”). The dimension of Emotional Awareness of Self may thus be especially important in moments that the mother is dealing with difficult emotions. The more awareness of the self in these moments, the better the mother may be able to stay present with and attentive with the child, and respond with a positive facial expression if the child smiles at the mother.

In the final model predicting mother’s positive facial expression, the IM-P subscale Compassion for the Child was the only predictor. The outcome mother’s positive facial expression measures mother’s the mother smiles at her child, but in contrast to the outcome dyadic synchrony of positive facial expression, mother’s positive facial expression is not about the mother and child reacting with smiles to each other’s smiles, but about the mother smiling at her child irrespective of the facial expression that the child shows. Compassion for the Child refers to an inner attitude of acceptance, openness, and patience the mother has for the child, especially when the child is experiencing difficulty. The relation between Compassion for the Child and positive facial expression shows that the inner attitude of acceptance, openness, and patience that is inherent to the experienced compassion that is reported by the mothers, translates to an important form of positive non-verbal communication to the child. Mother’s positive facial expressions are essential in the development of emotional co-regulation between mother and child, and thus in the development of child emotion regulation (Aktar et al. 2017). In the current study, both mother’s positive facial expression (irrespective of the child’s emotional expression) and coordination of positive facial expression (co-occurrence of positive facial expressions) was included as an outcome measure. It is not surprising that only the first was associated with Compassion for the Child, as compassion typically applies to situations that the child may not be feeling happy. The current study shows that mothers with higher self-rated Compassion for the Child, are more able to show their emotional availability to their child by keeping their facial expression positive, also when the child is not smiling at them.

Compassion for the Child was also a significant predictor of maternal sensitivity (and a borderline significant predictor of maternal acceptance of the child). Sensitivity and acceptance are two aspects of parental behavior which may represent two different sides of what compassion for a child may encompass. The inner attitude of being compassionate may translate itself in the first place in being able to stay present, listen to, and care for the child. This overlaps with what the acceptance of the child aims to measure, namely the extent to which mothers react either in a loving and accepting or in a rejecting manner to their child, and to the child’s emotions and initiatives. But a compassionate attitude may also translate more into compassionate action. The main characteristics of sensitivity are the ability to perceive the signs that the child gives the mother, to understand the underlying needs of the child, and to respond accordingly. Thus, being compassionate does not only refer to an inner attitude, but also the willingness to put this inner attitude into practice. A compassionate mother is open to recognize difficulties the child may be experiencing, and to give the proper support, or fulfill the child’s needs as well as possible.

The last aspect of maternal behavior that could be predicted significantly by dimensions of MP was non-attuned mind-related comments. Mothers who scored themselves as better at Listening with Full Attention, made less mistakes in “reading” their child’s mind. Listening with Full Attention refers to the ability to stay present with the child, without getting distracted, or being too busy or hurried to be able to pay attention and listen to the child. Possibly, staying present in the current moment with the child, and really listening to them, helps parents in understanding how the child may be feeling or what the child may be thinking. When the mother is consumed with her own thoughts and feelings (for example worries, memories, likes, and dislikes) chances are higher that this will impact the assumptions she will make about the child’s state of mind (Bögels et al. 2010).

Emotional Awareness of the Child, Non-judgmental Acceptance of Parental Functioning, and Emotional Non-reactivity in Parenting were not significantly related to observed parenting behaviors or the parent-child interaction quality. Non-judgmental Acceptance of Parental Functioning refers to the inner attitude (non-judging, and with acceptance) that parents have in relation to themselves instead of to their children, therefore, it is not surprising that no significant associations with the outcome measures were found. It is more surprising that there is no significant relation between Emotional Awareness of the Child and the outcomes, because awareness of how the child may be feeling, may be a prerequisite for being able to react sensitively, being accepting towards the child, and being able to comment on the child’s thoughts and feelings. Possibly, it is difficult for mothers to self-report on how accurate their Emotional Awareness of the Child is. The last IM-P subscale that did not show significant associations with any of the outcomes was Emotional Non-reactivity in Parenting, which measures the extent to which parents emotionally overreact to their children (or the extent to which they succeed in inhibiting overreactivity). It was shown that the subscale Emotional Non-reactivity in Parenting had a moderate to strong negative correlation with the overreactivity subscale of the Parenting Scale (De Bruin et al. 2014), and, in turn, the overreactivity subscale of the Parenting Scale correlated significantly with observed overreactivity (Arnold et al. 1993). The current study cannot answer the question whether the IM-P subscale Emotional Non-reactivity in Parenting also correlates with observed overreactivity, as this was not directly measured. Overreacting is related bidirectionally to complicated child behavior (Del Vecchio and Rhoades 2010). A 10-min unstructured play-situation, used for the video-observation in the current study, may not trigger complicated child behavior. Therefore, these situations may not have been ecologically valid to observe overreactive parenting behavior (Arney 2004). If a lot of parental overreactivity would have been seen, this might have for example affected the scale Acceptance of the child. Another possible explanation for the fact that Emotional Non-reactivity in Parenting did not add to the explained variance of the outcomes, is that the scale may be less suitable for parents of young children. Internal consistency of this subscale was also lower in the current study than in the validation study that included older children and adolescents, and some items were deleted to improve reliability in the current study group.

### Limitations and Future Research

The current study had several limitations. A large proportion on the data of some of the outcomes (emotional communication/dyadic synchrony) was missing, which may have negatively influenced power for these specific outcomes. Another limitation was the generalizability of the results. Although this study has a clinically representative population, the results cannot be generalized to all parents. Another limitation was that some of the IM-P subscales showed insufficient reliabilities. It may be that not all items are appropriate for the age category that we included in the current study. Strengths of the current study were the fact that it made use of relatively objective outcome measures, and the wide variety of outcome measures that were used. Future research could examine the question which adjustments of the IM-P are needed for parents with infants and toddlers. It could also be studied which factors, such as depression, influence the parental perception of their own MP. Furthermore, a more comprehensive understanding of the value of self-reported MP could be gained by studying the relation between the IM-P and the way in which a child reacts to the parent, and thus include additional constructs such as observed child behavior or emotional communition. Also, the value of partner-perceived mindful parenting in the prediction of quality of parenting behavior or the parent-child interaction could be explored, or whether it is possible to develop an observational measure of MP. Finally, it would be interesting to examine if a change in MP, for example, after following a MP training, is associated with a change in observed parental behavior and parent-child interaction.

Overall, the current study advances preliminary evidence on the relation between the IM-P total score and several dimensions of maternal parenting and parent-child interaction in mothers with young children who seek a Mindful with your baby/toddler training because they experience difficulties in caregiving. The IM-P total score was related to an outcome that represents the attention for, and presence with the child of the mother, namely mother’s gaze to the child. Furthermore, specific dimensions of MP were predictive of specific aspects of maternal behavior or the mother-child interaction. For example, mothers who self-rated high on Listening with Full Attention made less mistakes when they made mind-related comments to their child, and mothers who self-rated high on Compassion for the Child were more sensitive to their child, and displayed positive facial expressions more often than mothers who scored lower on Listening with Full Attention or Compassion for the Child, respectively. When the IM-P is administered with the aim to gain understanding on the quality of parenting, it is important to not only look at the results of the IM-P total score, but to attend to the outcomes of the different subscales.
